# Identification of two regulatory binding sites which confer myotube specific expression of the mono-ADP-ribosyltransferase *ART1 *gene

**DOI:** 10.1186/1471-2199-9-91

**Published:** 2008-10-21

**Authors:** Maik Friedrich, Levin Böhlig, Ralf D Kirschner, Kurt Engeland, Sunna Hauschildt

**Affiliations:** 1 Institute of Biology II, Dept. of Immunobiology, University of Leipzig, Talstraße 33, D-04103 Leipzig, Germany; 2 Molecular Oncology, Dept. of Obstetrics and Gynaecology, University of Leipzig, Semmelweisstr. 14, D-04103 Leipzig, Germany; 3 Interdisciplinary Centre for Clinical Research (IZKF) Leipzig, Semmelweisstr.14, D-04103 Leipzig, Germany

## Abstract

**Background:**

Mono-ADP-ribosyltransferase (ART) 1 belongs to a family of mammalian ectoenzymes that catalyze the transfer of ADP-ribose from NAD^+ ^to a target protein. ART1 is predominantly expressed in skeletal and cardiac muscle. It ADP-ribosylates α7-integrin which together with β1-integrin forms a dimer and binds to laminin, a protein of the extracellular matrix involved in cell adhesion. This posttranslational modification leads to an increased laminin binding affinity.

**Results:**

Using C2C12 and C3H-10T 1/2 cells as models of myogenesis, we found that ART1 expression was restricted to myotube formation. We identified a fragment spanning the gene 1.3 kb upstream of the transcriptional start site as the functional promoter of the *ART1 *gene. This region contains an E box and an A/T-rich element, two conserved binding sites for transcription factors found in the promoters of most skeletal muscle specific genes. Mutating the DNA consensus sequence of either the E box or the A/T-rich element resulted in a nearly complete loss of *ART1 *promoter inducibility, indicating a cooperative role of the transcription factors binding to those sites. Gel mobility shift analyses carried out with nuclear extracts from C2C12 and C3H-10T 1/2 cells revealed binding of myogenin to the E box and MEF-2 to the A/T-rich element, the binding being restricted to C2C12 and C3H-10T 1/2 myotubes.

**Conclusion:**

Here we describe the molecular mechanism underlying the regulation of the *ART1 *gene expression in skeletal muscle cells. The differentiation-dependent upregulation of ART1 mRNA is induced by the binding of myogenin to an E box and of MEF-2 to an A/T-rich element in the proximal promoter region of the *ART1 *gene. Thus the transcriptional regulation involves molecular mechanisms similar to those used to activate muscle-specific genes.

## Background

Mono ADP-ribosytransferases (ARTs) are an important class of enzymes that catalyse the transfer of the ADP-ribose from NAD^+ ^to a specific amino acid residue in the target protein [1,2]. This reaction has been originally identified as the pathogenic mechanism of bacterial toxins, including cholera, pertussis and diphtheria toxin [3,4]. There is increasing evidence that endogenous ARTs also play important roles in higher animals and human [5-7]. So far, the family of mammalian ARTs comprises five members (ART1-5) [8]. They are all ectoenzymes, anchored in the outer leaflet of the plasma membrane via a glycosylphosphatidylinositol-tail with the exception of ART5 which is secreted to the extracellular space [9]. Among the five ARTs, only ART1, ART2 and ART5 exhibit enzyme activity whereas ART3 and ART4 appear to have lost their catalytic activity [8,10]. ART1 was identified as the first mammalian ART after protein purification from skeletal muscle and cDNA cloning from rabbit [11], human skeletal muscle [12] and mouse lymphoma cells [13]. ARTs show a rather tissue-specific expression with ART1 predominantly expressed in skeletal and cardiac muscle [8,11,14]. ART3 and ART5 besides being abundant in testis [8,14] are also expressed in muscle tissues.

In search of proteins being ADP-ribosylated by ART1 α_7_-integrin was identified as a key protein [15,16]. α_7_-integrin is expressed specifically in skeletal and cardiac muscle, forms a dimer with β_1_-integrin, and binds to laminin, an extracellular matrix protein [17,18]. ADP-ribosylation has a positive effect on the interaction of α_7_/β_1_-integrin with its ligand, laminin [19]. It may represent a mechanism of upregulation of α_7_/β_1-_integrin function in situations where enhanced interactions are required such as muscle injuries or diseases [20].

Interestingly in mouse skeletal muscle cells, the expression of ART1 correlates with the transition from nondifferentiated mononucleated myoblasts to multinucleated nonreplicating myotubes [15]. This step is controlled by a tightly regulated transcriptional program that involves two key transcription factor families: the basic helix-loop-helix protein (bHLH) family [21-24] consisting of MyoD, Myf5, myogenin and MRF4 and the myocyte enhancer binding factor 2 (MEF-2) family of the MADS-box factors [25,26].

MEF-2 factors synergize with myogenic bHLH proteins to regulate transcription and myogenesis. The DNA binding recognition sequence of the bHLH proteins is an E box, which is located in the regulatory regions of many muscle-specific genes [27-29]. MEF-2 proteins bind as homo- and heterodimers to an A/T-rich DNA consensus also found in the promoter regions of nearly every known muscle-specific gene [30].

When analysing the promoter sequence from the *ART1 *gene we found putative myogenin and MEF-2 binding sites, suggesting that these factors may participate in the regulation of the *ART1 *gene. Using C2C12 and C3H-10T 1/2 cell lines, we show that activation of the *ART1 *promoter is associated with differentiation of myoblasts into myotubes. Mutation and deletion analysis of the promoter revealed that myogenin and MEF-2 binding sites are necessary and sufficient for transcriptional activity of the promoter. Here we report for the first time that transcription factors activating the promoter of most skeletal muscle specific genes regulate transcription of ART1, an enzyme involved in posttranslational modification of adhesion proteins.

## Methods

### Material

Oligonucleotides were synthesized by Invitrogen GmbH (Karlsruhe, Germany). Restriction enzymes (DpnI, XhoI, BglII) and the exo-Klenow enzyme were obtained from Fermentas GmbH (Saint Leon-Rot, Germany) and Pure Yield™ Plasmid Mini Preparation System, pRL4.70 plasmid, pGL4.10 basic plasmid and the dual luciferase reporter assay were purchased from Promega (Madison MA, USA). The iQ™ SYBR^® ^Green Supermix and the Bradford assay were from Bio-Rad Laboratories GmbH (München, Germany), poly(dIdCxdIdC) and etheno-NAD^+ ^were from Sigma (Taufkirchen, Germany).

### Cell culture

The mouse C3H-10T 1/2 mesenchymal progenitor cell line and stably MyoD transfected C3H-10T 1/2 cells, here designated as MyoD-myoblasts, were obtained from Dr. A. Buchberger (University of Braunschweig, Germany). Cells were cultured in growth medium (37°C, 5% CO_2_) consisting of Dulbecco's modified Eagle's medium (DMEM), supplemented with 2 mM L-glutamine, 4.5 g/l D-glucose, 100 U/ml penicillin, 100 μg/ml streptomycin and 10% fetal bovine serum (FBS). The mouse C2C12 cell line (a kind gift from Dr. Dr. T. Braun, Max-Planck-Institute for Heart and Lung Research, Bad Nauheim, Germany) was cultured in the same growth medium containing 20% instead of 10% FBS. Proliferating myoblasts (C3H10T 1/2-MyoD cells and C2C12 cells) were grown at low density to prevent differentiation.

Fusion of myoblasts into myotubes was induced by growing cells to 80% confluence before replacing growth medium by fresh medium supplemented with 2% horse serum (HS) instead of FBS or by growing cell at confluence for several (5 up to 7) days in growth medium.

### RNA isolation and reverse transcription

Total RNA was isolated from C3H-10T 1/2- and C2C12 cells (progenitors, MyoD-myoblasts, myoblasts, myotubes) and from quadriceps skeletal muscles (CBA mouse) using the RNeasy mini kit (Qiagen, Hilden, Germany) according to the manufacturer's instruction. First-strand cDNA was synthesized using 4 μg of total RNA in a 20 μl reverse transcripton reaction mixture as described previously [31].

### Polymerase chain reaction (PCR)

PCR reactions were performed as described previously [10,32] with minor modifications. In brief, the following primer pairs were used: mART1-full-UTR-fwd 5'-ATC CCA GGA GAC CAG GTC AG-3' and mART1-full-rev 5'-GCA CAG TTG ACC AGC CTT CT-3'; β2 m fwd: 5'-CTG ACC GGC CTG TAT GCT AT-3' and β2 m rev: 5'-TTT TCC CGT TCT TCA GCA TT-3'. Amplification of ART1- and beta-2 microglobulin (β2 m) mRNA was carried out at 61°C, 30 cycles and 60°C, 25 cycles respectively. The PCR products were separated by electrophoresis on 1.8% agarose gels (FMC Bioproducts, Rockland, MA, USA) containing 1.25 μg/ml ethidium bromide and visualized under ultraviolet light. The 100 bp ladder (Invitrogen GmbH, Karlsruhe, Germany) served as a standard.

### Semi-quantitative real-time PCR

The reaction mixture contained 10 μl of the iQ™SYBR^® ^Green supermix, 125 nM forward and reverse primers and 1 μl of cDNA template in a final volume of 20 μl. The list of intron-spanning primers amplifying ART1, ART3 and ART5 cDNA is shown in Table 1. Samples were run in duplicate in a 7300 real time PCR cycler system (Applied Biosystems, Darmstadt, Germany). The reactions were performed under the following conditions: initial denaturation at 95°C for 3 min, followed by 39 cycles of 15 s denaturation at 95°C, 25 s of primer annealing 60°C (except for ART5 – 61°C) and 28 s of extension/synthesis at 72°C. Product quantification was optimal at 72°C, except of ART1 (28 s at 83°C). Negative controls were performed with total RNA or water as template. Following PCR, the melting curve for each product was determined and the correct size calculated by agarose gel analysis. All cDNA products were confirmed by sequencing to check their identity. Calculations were carried out as described previously [33].

**Table 1:** List of intron-spanning primers used in real-time RT-PCR

**target mRNA**	**primer name**	**oligonucleotides sequence (5' → 3' orientation)**
mono ADP-ribosyltransferase 1 (ART1)	ART1-rt_fwd ART1-rt_rev	CAG GGG CTA CTC CTT TTT CC CCC AGA CCT GCA CTT CTT TT
		
mono ADP-ribosyltransferase 3 (ART3)	ART3-rt_fwd ART3-rt_rev	AAT GGT CAC CAC GCT GCT CCC TCT TCA TCT GCG GAA TA
		
mono ADP-ribosyltransferase 5 (ART5)	ART5-rt_fwd ART5-rt_rev	TGT GTC CTC AAG AGC AGT CG CA ACT CTG GTT GGA CAG GT
		
hypoxanthine phosphoribosyltransferase 1	Hprt1_fwd Hprt1_rev	CCA ACT TTG CTT TCC CTG GT CTG GCC TGT ATC CAA CAC TTC
		
beta-2-microglobulin	β2m_fwd β2m_frev	CTG ACC GGC CTG TAT GCT AT TTT TCC CGT TCT TCA GCA TT

### Treatment of cells with etheno-NAD^+^

C3H-10T 1/2 mesenchymal progenitors, MyoD-myoblasts and myotubes were harvested from 6 well cell culture plates by incubating cells with PBS/EDTA at 4°C. After washing cells were incubated in the presence or absence of 100 μM etheno-NAD^+ ^for 30 min at 37°C, followed by subsequent washing with PBS containing 10% Haemaccel^® ^(Hoechst, Frankfurt, Germany) and 0.1% sodium azide. Etheno-ADP-ribosylated proteins on the cell surface were measured by FACS analysis as described previously [10]. The anti etheno-adenosine specific antibody 1G4 (IgG2a) [34] was kindly provided by Dr. F. Koch-Nolte (University Medical Center Hamburg, Germany).

### Luciferase reporter constructs

Genomic DNA was extracted from mouse C3H10T 1/2 cells. Two fragments of the *ART1 *promoter (pART1-L: -1236/+37 and pART1-L: -322/+37 respective to the transcription start site) were amplified by PCR using the upstream primers (-1236) luc-pART1-L-fwd: 5'-TTT TTT **CTC GAG **TTC TCT CGC ACC TCC CTT GT-3', (-322) luc-mART1-S-fwd: 5'-TTT TTT **CTC GAG **CCA CCA CCA CCA CAG AAC AA-3' (both tagged with a XhoI restriction site, shown underlined) and (+37) luc mART1-rev2: 5'-TTT TTT **AGA TCT **ATG GCG GGC AAA GCT GAC CT-3' (tagged with a BglII restriction site, shown in bold letters). Both products were inserted in the promoter-, enhancerless pGL4.10 basic *firefly *luciferase reporter plasmid (Promega, Madison MA, USA). Recombinant plasmids were isolated with the Pure Yield™ Plasmid Mini Preparation System (Promega, Madison MA, USA) and sequenced to check their identity.

### Site-directed mutagenesis of the pGL4.10-pART1-S reporter construct

The pGL4.10-pART1-S construct was used as a template in the QuickChange^® ^site-directed mutagenesis procedure according to the manufacturer's instructions (Stratagene, La Jolla, CA, USA). Forward and reverse primers used to create the point mutations in the wild type E box and A/T -rich element were: mART1-mut-E-box fwd: 5'-CAC ATT TCT GAG **tg**G CT**c **TGT GGC GAC AGC AGG-3', mART1-mut-E-box rev: 5'-CTG TCG CCA CA**g **AGC **ca**C TCA GAA ATG TGA CAC-3'; mART1-mut-A/T-rich fwd: 5' CAA GGA GAC AG**a g**TG AAc **ac**G ACG AGT GTC ACA TTT C-3' and mART1-mut-A/T-rich rev: 5'-GTG ACA CTC GTC **gtg **TTC A**ct **CTG TCT CCT TGT CAA G-3'. Sequences shown in bold lower case represent mismatch bases introduced to obtain the desired mutation. Following amplification, the reaction mixture was treated with DpnI to eliminate the template DNA. Each of the mutated amplification products was transformed into the *Escherichia coli *DH 5α strain. After having confirmed the desired mutations in the mutated promoter sequence the mutated promoter insert was subsequently cloned into a new pGL4.10 plasmid to ensure that no unwanted mutations occurred in plasmid backbone.

### Transient transfection and luciferase reporter gene assays

All transfections were carried out with FuGENE6^®^HD (Roche, Mannheim, Germany) according to the manufacturer's instructions. For promoter reporter assays C3H10T 1/2-, C3H10T 1/2-MyoD- and C2C12 cells were seeded at 2 × 10^4 ^cells/0.5 ml growth medium in a 24-well cell culture plate. To induce differentiation of myoblasts into myotubes, C3H10T 1/2-MyoD and C2C12 cells were seeded at 2 × 10^5 ^cells/0.5 ml growth medium. Cells were cotransfected with 400 ng pGL4.10 vector or 400 ng promoter-reporter construct and 30 ng promoterless pGL4.70 (Promega, Madison MA, USA) per well, respectively. Each transfection was carried out in triplicate. 20 h after transfection the medium was replaced by fresh growth medium or differentiation medium (to induce differentiation into myotubes). Dual Luciferase Reporter Assays were carried out 72 h after transfection in triplicate according to the manufacturer's instructions. The *firefly *luciferase activity was normalized to *renilla *luciferase activity in order to compensate variations in transfection efficiencies. The activity of the promoter-reporter constructs is given as fold induction compared with the activity of the pGL4.10 promoterless plasmid.

### Electrophoretic mobility shift assay (EMSA)

DNA probes from the *desmin *gene E box, the *N-10 *gene A/T-rich element and the *ART1 *gene E box and A/T-rich element (wt/mut) were generated by annealing the forward oligonucleotides listed in table 2 with the respective reverse oligonucleotides. Fill-in labeling reactions were carried out with [α-^32^P]dCTP (MP Biomedicals GmbH, Eschwege, Germany) using the exo-Klenow enzyme.

**Table 2:** List of forward oligonucleotides used for electrophoretic mobility shift assays (EMSA).

**target name**	**element**	**wt/mut**	**forward oligonucleotide sequences (5' → 3' orientation)**
**ART1**	**E box **(-80/-54)	**wt**:	gg CAT TTC TGA G**CA GCT G**TG TGG CG ACA
		**mut**:	gg CAT TTC TGA G***TG ***GCT ***C***TG TGG CG ACA
	**A/T-rich **(-169/-82)	**wt**:	gg AGG AGA CAG **CTT GAA ATA G**AC GAG TGT C
		**mut**:	gg AGG AGA CAG ***AG***T GAA ***CAC ***GAC GAG TGT C
**Desmin**	**E box **(-845/-820)	**wt**:	gg TTC GCC TTG G**CA GCT G**TT GCT CGT AG
**N-10**	**A/T-rich**	**wt**:	gg AGG AAA A**CT ATT TAT AG**A TCA AAT

Putative consensus sequences are indicated by bold letters. Positions of exchanged nucleotides in mutated (mut) DNA probes are in italic, bold letters. Lower case 'gg' denotes two added nucleotides used for fill-in labeling reactions.

C3H-10T 1/2 (Pg, MyoD-Mb, Mt) and C2C12 (Mb, Mt) cells were washed with PBS, scraped into ice cold PBS and centrifuged. Cell pellets were incubated 20 min at 4°C in lysis buffer (10 mM Hepes-KOH, pH 7.9, 1.5 mM MgCl_2_, 10 mM KCl, 0.5 mM dithiothreitol (DTT), 0.5 μg/ml leupeptin, 0.5 μg/ml vanadat, 1 μg/ml pepstatin, 1 μg/ml aprotinin and 1 μg/ml NaF) and subjected to vigorous vortexing. Nuclei were collected and incubated with extraction buffer (20 mM Hepes-KOH, pH 7.9, 10% glycerol, 420 mM NaCl 1.5 mM MgCl_2_, 10 mM EDTA, 0.5 mM dithiothreitol (DTT), 0.5 μg/ml leupeptin, 0.5 μg/ml vanadat, 1 μg/ml pepstatin, 1 μg/ml aprotinin and 1 μg/ml NaF) for 20 min at 4°C and subsequently sonicated for a few seconds.

Clarified extracts were aliquoted and stored at -80°C. Ten to fourteen micrograms of nuclear proteins were incubated with binding buffer (10 mM Tris, pH 7.5, 40 mM KCl, 0.5 mM DTT, 10% glycerol) for 25 min at room temperature in the presence of 1 μg of poly(dIdCxdIdC) (Sigma-Aldrich Inc.) and 1 μl [α-^32^P]-labelled DNA probe (2 × 10^5 ^c.p.m.). To identify myogenin- or MEF2-containing complexes, 1 μg of mouse anti myogenin mAb (clone FD5, Dako Cytomation) or 1 μg of anti MEF-2 polyclonal antiserum (C-21, Santa Cruz Biotechnology) were added to the assay mixture. As a negative control 1 μg mouse IgG1 antibody (MOPC-21, Sigma-Aldrich Inc.) or rabbit polyclonal IgG antiserum (C-16, Santa Cruz Biotechnology) were used. Protein-DNA complexes were electrophoretically separated on 5% nondenaturing polyacrylamide gels at 10°C using 0.5 × TBE (A/T-rich elements probes) or 1 × TBE (E box probes) as running buffer. Gels were dried and labelled complexes were visualized by a Phosphoimager system (Fuji).

### Chromatin immunoprecipitation (ChIP) assays

ChIP-assays were carried out using C2C12 cells (Mt, Mb) according to [35] with minor modifications: magnetic Dynabeads^® ^coupled with protein-G (Invitrogen GmbH, Karlsruhe, Germany) were used instead of agarose beads and the purification of DNA fragments was carried out with the PCR-purification kit (Qiagen, Hilden, Germany) instead of a chloroform/phenol extraction procedure. Protein crosslinks were precipitated using 5 μg of a rabbit polyclonal anti myogenin antiserum (M-225, Santa Cruz Biotechnology), a rabbit polyclonal anti MEF-2 polyclonal antiserum (C-21, Santa Cruz Biotechnology) or a rabbit polyclonal IgG antiserum (nr.-31450, Thermo Scientific).

Samples were analysed by PCR as described previously [10,32] using following primers: Chip-HPRT-1-fwd: 5'-CTG CCT CTG CCT CCT AAA TG-3', Chip-HPRT-1-rev: 5'-TGT CGT CTC CCA GAG GAT TC-3'; Chip-Desmin-enhancer-fwd: 5'-TCA GCC TTC CTT GAC ACC TC-3', Chip-Desmin-enhancer-rev: 5'-ACA CCA CGG GTT TGT GTT TT-3'; Chip-mART1-fwd: 5'-GGG GTG ATC AGA GTC CAG AA-3', Chip-mART1-rev: 5'-GGA GGG CAG CTT TCT CTT TT-3'. Product amplification was carried out at 60°C with 29 cycles using 1 μl precipitated DNA solution as template.

### Data analysis

Following programs were used: the standard nucleotide-nucleotide BLAST [blastn] program [36] to screen genome databases; Neural Network Promoter Prediction (NNPP), available at BCM Search Launcher, to predict the *ART1 *promoter region and transcriptional start site; CLUSTAL W software [37] for multiple amino acid sequence alignment, and the TESS program (Transcription Element Search System) to predict transcription factor binding sites and to browse the TRANSFAC database [38].

## Results

### Expression of ART1, ART3 and ART5 mRNA in myogenic cell lines during skeletal muscle differentiation *in vitro*

Among the five mammalian ARTs, ART1, ART3 and ART5 have been shown to be expressed in skeletal muscle [8]. Based on the finding that ART1 was absent from C2C12 mouse myoblasts but appeared with differentiation of myoblasts to myotubes [15] it was of interest to determine whether ART3 and ART5 followed a similar expression pattern.

**Figure 1:**
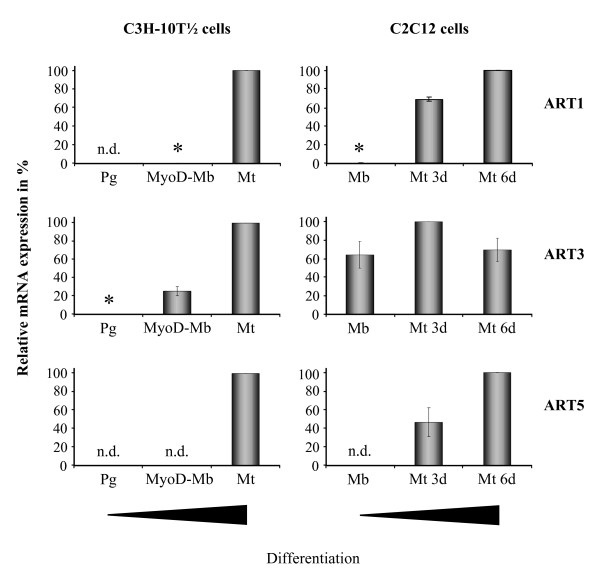
**Real-time RT-PCR analysis of ART1, ART3 and ART5 mRNA expression in myogenic cell lines during skeletal muscle differentiation *in vitro***. The mRNA of C3H-10T 1/2 and C2C12 cells at different stages of differentiation (progenitors (Pg), MyoD-myoblasts (MoD-Mb), myoblasts (Mb), myotubes (Mt)) was isolated and mRNA levels of ART1, ART3 und ART5 were measured by semiquantitative real-time RT-PCR. Relative mRNA levels were standardized to the expression of beta-2 microglobulin housekeeping gene and mRNA concentrations from C3H-10T 1/2 cells (Mt) and C2C12 cells (Mt 6d, ART3 – Mt 3d) were set as the 100% reference. Asterisks indicate values less that 0.3%. Data are means ± S.D. of three independent experiments. n.d.: not detectable.

Real time RT PCR was carried out to measure ART1, 3 and ART5 mRNA in the cell lines C3H-10T 1/2 and C2C12 at various stages of differentiation. As seen in Fig. 1 all three mRNAs are expressed in C2C12 and C3H-10T 1/2 myotubes. They are neither detectable in the C3H-10T 1/2 mesenchymal progenitor cells nor, with the exception of ART3 mRNA, in C2C12 and C3H-10T 1/2 myoblasts. Thus ART5 similar to ART1 mRNA is strongly upregulated during muscle cell differentiation. Upregulation of ART3 mRNA was only observed in C3H-10T 1/2 cells.

### ADP-ribosylation of cell surface target proteins during muscle cell differentiation is restricted to the myotube stage

**Figure 2:**
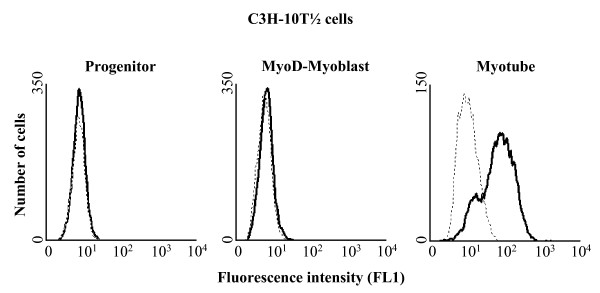
**Etheno-ADP-ribosylation of cell-surface proteins on C3H-10T1/2 cells**. C3H-10T 1/2 cells at different stages of differentiation (progenitors, MyoD-myoblasts, myotubes) were incubated for 30 minutes in the absence (dashed lines) or presence (solid lines) of 100 μM etheno-NAD. Cells were then washed, incubated with the primary monoclonal antibody 1G4, stained with a FITC-conjugated goat-anti-mouse secondary antibody and subjected to FACS analysis. Data show one representative experiments out of three.

In previous studies Zolkiewska *et al. *[15] showed that mono ADP-ribosylation of cell surface target proteins of intact C2C12 cells was restricted to differentiated myotubes. To test whether this also applied to C3H-10T 1/2 cells we incubated progenitor cells, MyoD-myoblasts and myotubes with etheno-NAD^+^, an NAD^+ ^analogue that contains an ethenoadenosine moiety. The etheno-ADP-ribosylated cell-surface proteins were visualized by flow cytometry using an etheno-adenosine-specific monoclonal antibody (1G4). Fig. 2 shows that ADP-ribosylated proteins are only present on differentiated C3H-10T 1/2 myotubes whereas they are neither found on progenitor cells nor on the MyoD-myoblasts.

Among the three ARTs being expressed at the myotube stage ADP-ribosylation of surface proteins can only be attributed to ART1. ART3 lacks any detectable enzyme activity [8,10] and ART5 can be excluded because it is secreted instead of being cell attached [9].

To verify that ART3 in contrast to ART1 is enzymatically inactive we analysed HEK 293-T cells stably transfected with ART1 or ART3. Only on ART1-transfected cells ADP-ribosylated proteins were detected (data not shown).

### Structural and functional features of the putative *ART1 *promoter

**Figure 3:**
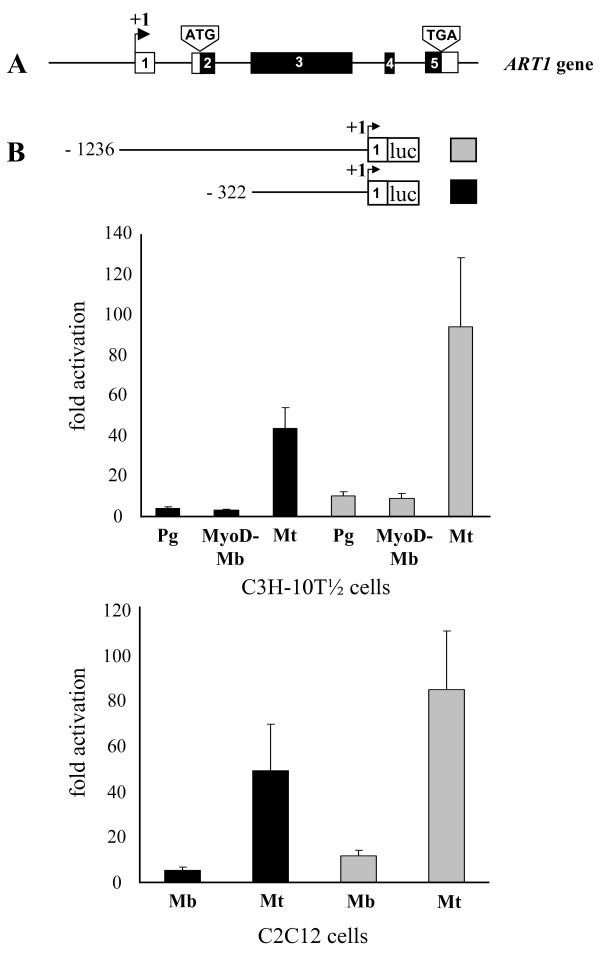
**The activity of a short and a long *ART1 *promoter fragment is upregulated during myotube formation**. (A) Shown is the exon-intron structure of the mouse *ART1 *gene as described previously [58]. Exons 1–5 are represented by boxes, introns by thin lines. Coding regions are indicated in black, non-coding regions in white. (B) A long (-1236/+36) and a short (-322/+37) fragment of the putative mouse *ART1 *promoter region (pART1-L: grey bars and pART1-S: black bars, respectively) was cloned into the pGL4.10 *firefly *luciferase reporter plasmid. C3H-10T 1/2 (Pg, MyoD-Mb, Mt) and C2C12 (Mb, Mt) cells were transfected with the plasmids and promoter activities were determined using the dual luciferase reporter assays normalized to *renilla *luciferase activity. Promoter activities are shown as fold activation respective to the pGL4.10 basal activity (pGL4.10 = 1). The data represent means ± S.D. of three independent experiments in triplicate. An arrow indicates the transcription start site numbered with +1 and 'luc' boxed indicates the *firefly luciferase *gene.

In contrast to the biological function of ART1 nothing is known about the molecular mechanisms regulating *ART1 *gene expression in skeletal muscle cells. For this purpose we cloned and analysed the promoter of the *ART1 *gene (Fig. 3A).

To verify the structure and length of the 5'UTR of ART1 transcripts from C3H-10T 1/2- and C2C12 myotubes and skeletal muscle tissue we performed RT-PCR analysis with primers amplifying the full-length 5'untranslated region (UTR) according to full-length data base ESTs entries [e.g. GeneBank: BY709497, BY709074, BY703474, CA460063.1] (data not shown). Respective to the transcription start site (numbered with +1) a long (-1236/+37) and a short (-322/+37) fragment of the putative *ART1 *promoter region (Fig. 3B) were cloned into the pGL4.10 basic *firefly *luciferase reporter plasmid. After transfection into C3H-10T 1/2 (Pg, MyoD-Mb, Mt) and C2C12 (Mb, Mt) cells the promoter activities were determined using the dual luciferase reporter assays. The putative long- and the short *ART1 *promoter fragments only showed basal promoter activity in C3H-10T 1/2 progenitor cells, C3H-10T 1/2 MyoD-myoblasts and C2C12 myoblasts. However, the activities of both fragments were strongly upregulated after differentiation of the proliferating mononuclear myoblasts into non proliferating multinuclear myotubes (Fig. 3B). Although compared with the short fragment, the long fragment showed an increased promoter activity, the major cis-regulatory elements which mediate the myotube specific upregulation of the promoter activity are located within the (-322/+37) fragment.

**Figure 4:**
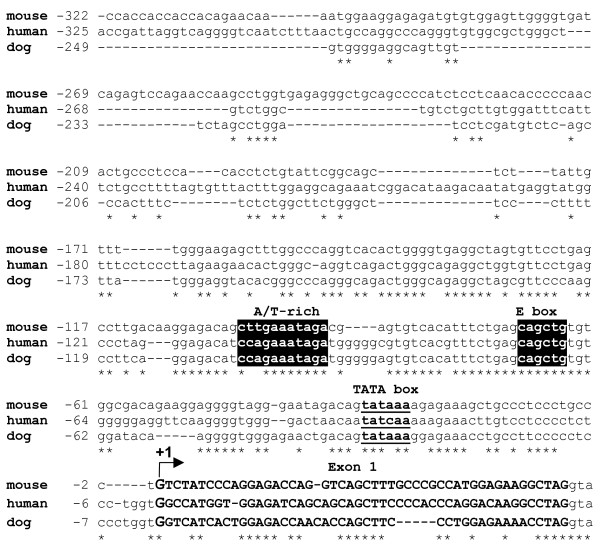
**Two conserved cis-regulatory consensus sites in the *ART1 *proximal promoter regions of mouse, human and dog are putative candidates for muscle specific gene expression**. Genomic sequences of the *ART1 *proximal promoter from human (NT_039433.7), mouse (NT_009237.17) and dog (AAEX02028845.1) were aligned using the CLUSTAL W software program [37] and analysed using the Transcription Element Search System (TESS) program [38]. The transcription start site (TSS) numbered with +1 is indicated by an arrow. The numbering of the nucleotides refers to the nucleotide position relative to the putative TSS. Conserved putative cis-elements are indicated by black boxes with sequences in white. A TATA box is shown in bold underlined lower case. The nucleotide sequence of exon 1 is shown in bold upper case, promoter and intron border in lower case. The symbol '-' indicates the lack of a corresponding sequence and '*' the identity between nucleic acids.

To identify conserved putative binding sites for transcription factors playing established roles in skeletal muscle cell development, the genomic sequences of the *ART1 *proximal promoter from mouse, human and dog were aligned using the CLUSTAL W software program [37] and analysed using the Transcription Element Search System (TESS) program [38]. Besides a classical TATA box motive, analyses revealed the existence of two conserved cis-regulatory consensus sites, an E box motive (5'-CAGCTG-3') and an A/T-rich element (5'-CTTGAAATAG-3') in the *ART1 *proximal promoter regions (Fig. 4).

### The E box and the A/T-rich element play a role in myotube-specific *ART1 *promoter activity

**Figure 5:**
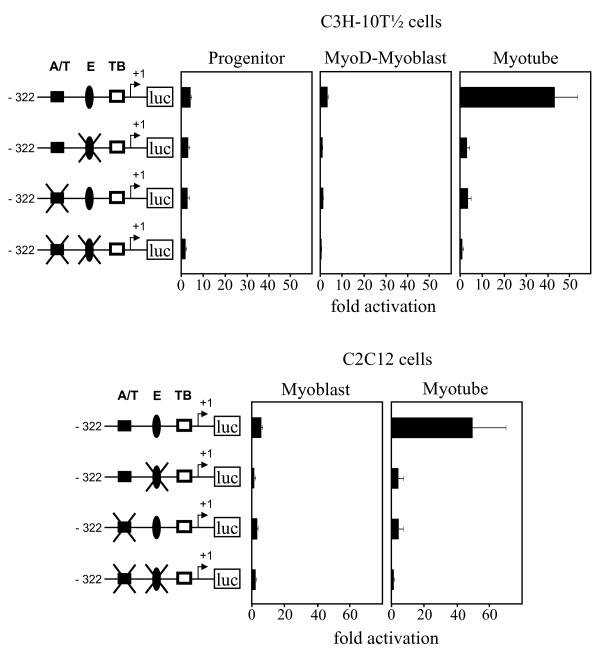
**An A/T-rich motif and an E box mediate the induction of the *ART1 *proximal promoter activity during myogenesis**. Candidate motifs for mediating muscle-specific expression of the mouse proximal *ART1 *promoter were altered by site-directed mutagenesis of the pART1-S (-322/+37) containing pGL4.10 plasmid. Cells at different stages of differentiation were transfected with the plasmids. Promoter activities were determined using the dual luciferase reporter assays normalized to *renilla *luciferase activity. Following symbols are used: black box – A/T-rich element (A/T); black oval – E box (E); white box – TATA box (TB) and 'luc' boxed – *firefly luciferase *gene. An arrow indicates the transcription start site numbered with +1. Mutations of symbolised A/T-rich element (A/T) and E box (E) are indicated by an X overlay. The data represent means ± S.D. of three independent experiments in triplicate.

To gain insight into the functional importance of the predicted regulatory motifs in directing myotube-specific promoter activity, the core consensus sequence of predicted sites was altered by mutagenesis of the parental mouse proximal *ART1 *promoter construct pART1-S (-322/+37). C3H-10T 1/2 (Pg, MyoD-Mb, Mt) and C2C12 (Mb, Mt) cells were transfected with the plasmids and promoter activities were determined. As seen in Fig. 5 the mutation of the (-64/-70) E box (5'-CAGCTG-3' to 5'-**TG**GCT**C**-3') as well as the mutation of the (-90/-100) A/T-rich element (5'-CTTGAAATAG-3' to 5'-**AG**TGAA**CAC**G-3') results in a 90% decrease in the promoter inducibility in myotubes compared with the parental wild-type construct. Double mutant shows residual promoter activity while the differentiation-specific induction is completely lost, indicating that indeed both motifs are of functional importance for myotube restricted *ART1 *promoter activation. As both motives are in close vicinity a cooperative role of the transcription factors binding to those motifs might be expected.

### Binding of myogenin to the proximal E box during muscle cell differentiation

**Figure 6:**
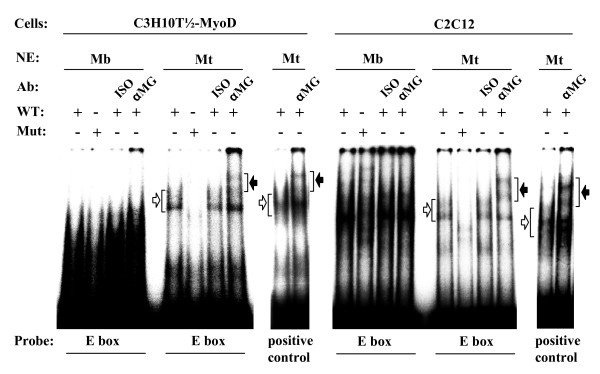
**Myogenin from myotube nuclear proteins extracts binds to the wild-type *ART1 *promoter E box**. Gelshift assays were performed by incubation of the [α-^32^P]dCTP labelled probe containing the E box (WT) of the *ART1 *gene promoter with nuclear protein extracts (NE) of C3H-10T 1/2 (MyoD-Mb, Mt) and C2C12 (Mb, Mt) cells. The specificity of the protein binding at the E box site was assessed by the use of a labelled probe containing a mutated ART1 E box sequence (Mut). For supershift assays, reaction mixtures were preincubated with antibodies (Ab) against myogenin (αMG) or with a non-specific isotype (ISO) as a negative control. Specific retarded protein/probe complexes are marked by white arrows and supershifted complexes by black arrows. As a positive control a probe containing a myogenin binding E box of the *desmin *gene promoter [43] was used.

Given the crucial role of the E box and the A/T-rich element in myogenic differentiation, we asked whether the functional properties of these sites are reflected by distinct DNA-protein interactions. Extensive analyses of skeletal muscle differentiation have shown that myogenic regulatory factors (MRFs) of the basic helix-loop-helix proteins (bHLH) family [21-24] are involved in skeletal muscle development. These bHLH factors (e.g. MyoD, Myf5, MRF-4, myogenin) bind as homo- or heterodimers with ubiquitously expressed E-proteins to a specific E box motive [39] in prominent muscle specific promoters and enhancers. Genetic studies indicate that MyoD and Myf5 are necessary to specify the skeletal muscle lineage [40] whereas myogenin appears to have a critical role in terminal differentiation of the specified muscle cells *in vivo *[41,42]. Because ART1 expression and the activity of the two cloned *ART1 *promoter fragments are restricted to differentiated non-proliferating multinuclear myotubes, myogenin was considered a favourite candidate to bind to the E box in the proximal *ART1 *promoter as a cis-regulatory factor. To test this hypothesis electrophoretic mobility shift assays (EMSA) were performed using the (-64/-70) E box from the *ART1 *proximal promoter as an EMSA probe (Fig. 6). As a positive control a probe containing a myogenin binding E box of the *desmin *gene promoter [43] was used.

As seen in Fig. 6 incubating nuclear extracts of C3H-10T 1/2- and C2C12 myotubes with the E box oligomer probe lead to the formation of a shifted complex that was not detectable in the presence of a mutated E box probe. The E box specific complex was supershifted by an anti-myogenin antibody but not by the corresponding isotype control confirming the specificity of the supershift. No supershift was observed when the experiments were carried out with nuclear extracts of myoblasts.

### Binding of MEF-2 to the A/T-rich element during muscle cell differentiation

Since the A/T-rich element in the *ART1 *proximal promoter is crucial for its activation as demonstrated by the luciferase reporter assay, we aimed to identify transcription factors that may bind to this site. According to the TESS program (Transcription Element Search System) myocyte enhancing factor 2 proteins (MEF-2), especially MEF-2A, are predicted to be candidates. MEF-2 binds to 5'-YTA(T/A)_4_TAR-3' [44] sequences in the regulatory regions of target genes. Although the sequence of the (-90/-100) *ART1 *A/T-rich element (5'-CTTGAAATAG-3') does not completely match the optimal consensus sequence it may bind MEF-2 protein.

**Figure 7:**
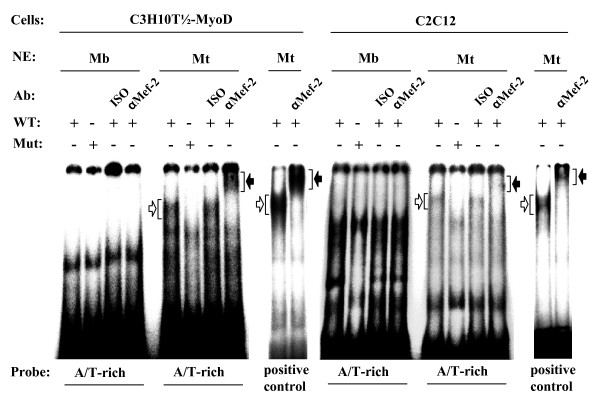
**MEF-2 from myotube nuclear protein extracts binds to the wild-type *ART1 *promoter A/T-rich element**. Gelshift assays were performed by incubation of the [α-^32^P]dCTP labelled probe containing the A/T -rich element (WT) of the *ART1 *gene promoter with nuclear protein extracts (NE) of C3H-10T 1/2 (MyoD-Mb, Mt) and C2C12 (Mb, Mt) cells. The specificity of the protein binding at the A/T-rich element was assessed by the use of a labelled probe containing a mutated ART1 A/T-rich sequence (Mut). For supershift assays, reaction mixtures were preincubated with antibodies (Ab) against MEF-2 (αMEF-2) or a non-specific isotype (ISO) as a negative control. Specific retarded protein/probe complexes are marked by white arrows and supershifted complexes by black arrows. As a positive control a probe containing an MEF-2 binding box of the *N-10 *gene promoter [25] was used.

To demonstrate binding of MEF-2 proteins to the A/T-rich site we performed gel-shift experiments using synthetic DNA probes of this element and nuclear extracts from C3H-10T 1/2- and C2C12 myotubes. As a positive control a probe containing a MEF-2 binding site of the *N-10 *gene promoter [25,45] was used. As shown in Fig. 7 nuclear extracts from C3H-10T 1/2- and C2C12 myotubes interacted with the oligomer probes whereas no specific complexes were formed when extracts from myoblasts or mutated oligonucleotide probes of the A/T-rich elements were used. Addition of an anti-MEF-2 antibody resulted in a supershift indicating that MEF-2 in fact interacts with the A/T-rich promoter region.

### Binding of myogenin and MEF-2 to the *ART1 *promoter during muscle cell differentiation *in vivo*

**Figure 8:**
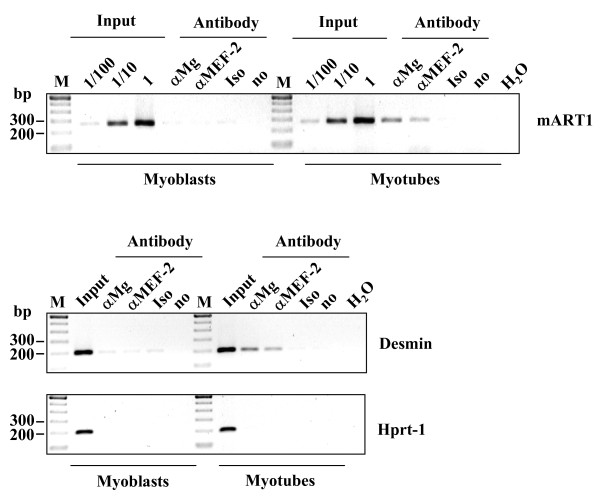
**Myogenin and MEF-2 bind to the *ART1 *promoter in myotubes, but not in myoblasts *in vivo***. The chromatin from C2C12 cells (myoblasts and differentiated myotubes) was cross-linked with formalin. Cells were lysed, the nuclear extracts were prepared and sonicated (four times 15 strokes; output, 70%; duty cycle, 60%; Bandelin Sonopuls GM70, Bandelin, Berlin, Germany). After precipitation with antibodies against myogenin (αMG), MEF-2 (αMEF-2) or with an unspecific IgG isotype antibody (Iso) as a control, the *ART1 *promoter region containing the E box und the A/T rich element was amplified by PCR from the precipitated DNA. Lanes are input DNA (Input) in dilutions: (1/100) 1:100, (1/10) 1:10 und undiluted (1), no antibody (no), water control (H_2_O) and DNA ladder (M). The PCR amplification of the *desmin *gene promoter enhancer [43] which contains a functional E box and a functional MEF-2 binding site served as a positive control whereas the amplification of the proximately part of the *HPRT-1 *housekeeping gene promoter served as negative control.

To confirm that endogenous nuclear myogenin and MEF-2 bind to the proximal part of the *ART1 *promoter *in vivo*, chromatin immunoprecipitation (ChIP) assays were carried out with C2C12 myotubes and myoblasts. As seen in Fig. 8, both myogenin and MEF-2 bind to the *ART1 *promoter in myotubes, but not in myoblasts.

As a positive control the *desmin *gene promoter enhancer [43] that contains a functional E box and functional MEF-2 binding site and as a negative control the proximal part of the *HPRT-1 *housekeeping gene promoter were used. Whereas both factors bind to the *desmin *gene promoter enhancer in a myotube restricted manner, no binding to the *HPRT-1 *gene promoter was observed.

## Discussion

The discovery of eukaryotic mono-ADP-ribosyltransferases (ARTs) is fairly recent [46,47]. Although the chromosomal localization, genomic organization and expression of the five mammalian *ART *genes are well understood nothing is known about the transcriptional control of these genes. The fact that three of the five mammalian ARTs namely ART1, ART3 and ART5 are highly expressed in skeletal muscle tissue made us analyse their transcriptional regulation during muscle cell differentiation in more detail. We used C2C12 and C3H-10T 1/2 cells, which provide well-established and reproducible models of myogenesis [48,49]. We found that ART1 and ART5 expression was restricted to myotubes while ART3 mRNA was already present in myoblasts of both cell types but absent from C3H-10T 1/2 mesenchymal progenitor cells. In previous studies we showed that the *ART3 *promoter consists of three regions (pα, pβ and pγ) and that the pβ promoter is preferentially used in skeletal muscle [10]. In line with these findings we observed an almost exclusive usage of the pβ promoter in C3H-10T 1/2 myoblasts transfected with the transcription factor MyoD (data not shown) suggesting that the promoter is a direct or indirect target of MyoD.

When defining the 5'bounderies of the ART5 gene and comparing them to the 5'flanking sequences of the *ART1 *gene Glowacki *et *al. [14] found that the two genes overlap at their 5'ends. Since both genes are coexpressed in heart- and skeletal muscle and as shown here in non-proliferating multinuclear myotubes and since the muscle-specific transcription start site of the *ART5 *gene overlaps with the 5'end of the *ART1 *gene one could speculate that their expression is regulated by a common promoter or regulatory element. Interestingly in testis, which contains ART5 but not ART1 mRNA, the transcription start site of ART5 lies outside the *ART1 *gene [14].

To understand the myotube specific expression of the *ART1 *gene in more detail we cloned and analysed the *ART1 *promoter. We identified a fragment spanning the nucleotide sequence from position -322 to +37 respective to the transcription start site (+1) as a functional promoter. We identified an E box and an AT-rich element in the promoter region, two binding sites for transcription factors with established roles in skeletal muscle development. By distinct DNA-protein interaction we showed binding of the transcription factors myogenin to the E box and MEF-2 to the A/T-rich element *in vitro*. We confirmed the binding of these factors to the proximal part of the *ART1 *promoter *in vivo*.

Extending the fragment to -1236 resulted in an increase in promoter activity suggesting the existence of additional enhancer elements within the -3221/-1236 region. Indeed, by using bioinformatics tools we have identified four additional cis-regulatory consensus sites: an A/T-rich motif (-1208,-1198) and three E boxes ((-1096,-1088), (-841,-836) and (-755,-750)), which were only present in the long fragment of the mouse ART1 promoter [see Additional file 1]. These putative binding sites for MEF-2 and bHLH-factors are potential candidates for enhancing muscle specific gene expression. Such enhancer elements are frequently found in the promoter region of prominent muscle specific genes (such as the *desmin*-[50,51] and the *muscle creatin kinase *gene [52]).

In addition to myogenin the other members of the muscle regulatory factors (MRFs) family, Myf5, Mrf-4 and MyoD are also expressed in skeletal muscle and play distinct but overlapping roles in regulating muscle cell development [53]. MyoD for example when introduced into C3H-10T 1/2 mesenchymal progenitor cells is able to convert the cells into myoblasts and as shown here to increase ART3 mRNA expression. For ART1 to become expressed it requires differentiation of myoblasts to myotubes. This process, which is achieved by removing serum from the cell cultures or by increasing cell-cell contact results in the activation of MyoD dependent transcription factors like myogenin (cross activation) the latter being essential for ART1 activation. Together with myogenin, MEF-2 proteins function combinatorially to activate ART1 transcription. These data show that ART1 expression is regulated in a similar way as the majority of skeletal muscle genes, which also require the coordinated activity of members of the two families of transcriptional regulators, MRF and MEF-2.

The myocyte enhancer factor 2 (MEF-2) belongs to the MADS (MCM1, agamous, deficiens, serum response factor) family of transcriptions factors. MEF-2 homo- or heterodimers bind an A/T-rich DNA sequence (5'-YTA(T/A)_4_TAR-3') [44] within the regulatory regions of several muscle specific genes [30].

Although the A/T rich DNA sequence within the *ART1 *promoter (5'-CTTGAAATAG-3') does not reflect a perfect match with the MEF-2 consensus sequence, MEF-2 binds to the sequence in the *ART1 *promoter. It contains the nucleotides TG at position 3 and 4 instead of an AA or AT, an unusual nucleotide sequence for a MEF-2 binding site. In case of a perfect MEF-2 consensus sequence in the *ART1 *promoter a sequence (5'-C *T****AT(A)****AAA *TAG-3') would result that contains a TATA box. A TATA box within the MEF-2 binding site in the *ART1 *promoter would only be -64 nucleotides upstream of the classical TATA box (-30/-25) of the promoter. If this putative sequence was used, a premature transcription start or interference with binding of MEF-2 could result. In either case a successful expression of ART1 would be questionable.

MEF-2 and myogenic bHLH factors play key roles in skeletal muscle development [40-42,54]. Both factors physically interact as part of a combinatorial complex that results in synergistic activation of transcription and myogenesis [54,55]. Cooperative activation is facilitated by the close proximity and coordinated positioning of the binding sites of MyoD and MEF-2 on the same face of the DNA in most muscle-specific promoters [56]. Wright *et *al. (1991) [27] found that MEF-2 binding sites are frequently associated with functional E boxes bound by myogenin and that these sites are often positioned with precise spacing to allow both factors to bind DNA simultaneously, which likely promotes protein-protein interactions by these factors [56]. Here we show that the binding sites for myogenin and MEF-2 in the *ART1 *promoter are also located in close vicinity separated by a short spacer of 19 nucleotides, sufficient to allow both factors to bind to DNA simultaneously. Furthermore the finding that mutating either the E box or the A/T-rich element results in a 90% decrease in the promoter inducibility during differentiation and that a double mutant construct hardly shows any promoter inducibility implies a cooperative role of the two transcription factors in regulating ART1 transcription during myogenesis.

To exert its effects i.e. to ADP-ribosylated α_7 _integrin the ART1 protein requires NAD^+^. Under physiological conditions the concentration of free NAD^+ ^in the extracellular compartment is in the submicromolar range [57] too low to attain significant activities. An increase could occur as a consequence of cell lysis during an infection or tissue injury or due to membrane injuries as a result of metabolic and mechanical stress (reviewed in [20]). Once activated, ART1 ADP-ribosylates α_7_-integrin, which together with β_1_-integrin binds to laminin. As ADP-ribosylation leads to an increased ligand binding, it may represent a mechanism of upregulation of α_7_β_1_-integrin function *in vivo *in injured or diseased muscle upon loss of plasma membrane integrity and efflux of NAD^+ ^[20].

The data presented here demonstrate that expression of the *ART1 *gene during myogenesis is under control of myogenin and MEF-2, two transcription factors critical for the activation of many skeletal muscle specific genes. Having identified the *ART1 *promoter it will be of interest if genetic defects and polymorphisms in the *ART1 *gene are associated with degenerative muscle diseases.

## Conclusion

The ectoenzyme ART1 is predominantly expressed in skeletal and cardiac muscle. It ADP-ribosylates α_7_-integrin, a cell surface protein of myotubes, which has a positive effect on the interaction of the α_7_β_1 _dimer with its ligand laminin [19]. Using C2C12 and C3H-10T 1/2 cells as a model of myogenesis we found that activation of the *ART1 *gene was restricted to the myotube stage. We identified the *ART1 *promoter and showed that it is regulated by the two transcription factors MEF-2 and myogenin, which play an essential role during muscle cell differentiation in the regulation of muscle-specific genes. An understanding of the transcriptional regulation of the *ART1 *gene should facilitate a better understanding of the regulation of ART1 expression under physiological and pathological conditions.

## Authors' contributions

MF designed together with SH the study, performed the molecular biological procedures, PCR, luciferase reporter gene assays, electrophoretic mobility shift assays, chromatin immunoprecipitation (together with LB), sequence alignments, carried out the enzymatic experiments and helped to draft the manuscript. LB participated in carrying out chromatin immunoprecipitation assays, generated promoter mutants and was substantially involved in the analysis and interpretation of the data. RDK participated in carrying out electrophoretic mobility shift assays and the data analysis. KE was involved in the analysis and interpretation of the data. SH supervised and designed the study with essential contribution by MF, participated in its coordination, and wrote the manuscript. All authors read and approved the final manuscript.

## Supplementary Material

Additional file 1 **The long fragment of the mouse *ART1 *proximal promoter contains four additional cis-regulatory consensus sites, putative candidates for enhancing muscle specific gene expression**. Genomic sequences of the *ART1 *proximal promoter from mouse (NT_009237.17) were analysed using the Transcription Element Search System (TESS) program [39]. The numbering of the nucleotides refers to the nucleotide position -323 to -1236 relative to the putative transcription start site (numbered with +1). Putative cis-elements are indicated by black boxes with sequences in white. Click here for file

## References

[B1] Okazaki IJ, Moss J (1996). Mono-ADP-ribosylation: a reversible posttranslational modification of proteins. Adv Pharmacol.

[B2] Ueda K, Hayaishi O (1985). ADP-ribosylation. Annu Rev Biochem.

[B3] Aktories K (1991). ADP-ribosylating toxins.

[B4] Honjo T, Nishizuka Y, Hayaishi O (1968). Diphtheria toxin-dependent adenosine diphosphate ribosylation of aminoacyl transferase II and inhibition of protein synthesis. J Biol Chem.

[B5] Jacobson MK, Jacobson EL (1989). ADP-ribose transfer reactions: Mechanisms and biological significance.

[B6] Haag F, Koch-Nolte F (1997). ADP-Ribosylation in Animal Tissues: Structure, Function and Biology of Mono(ADP-Ribosyl)transferases and Related Enzymes.

[B7] Seman M, Adriouch S, Haag F, Koch-Nolte F (2004). Ecto-ADP-ribosyltransferases (ARTs): emerging actors in cell communication and signaling. Curr Med Chem.

[B8] Glowacki G, Braren R, Firner K, Nissen M, Kuhl M, Reche P, Bazan F, Cetkovic-Cvrlje M, Leiter E, Haag F (2002). The family of toxin-related ecto-ADP-ribosyltransferases in humans and the mouse. Protein Sci.

[B9] Okazaki IJ, Moss J (1998). Glycosylphosphatidylinositol-anchored and secretory isoforms of mono-ADP-ribosyltransferases. J Biol Chem.

[B10] Friedrich M, Grahnert A, Klein C, Tschop K, Engeland K, Hauschildt S (2006). Genomic organization and expression of the human mono-ADP-ribosyltransferase ART3 gene. Biochim Biophys Acta.

[B11] Zolkiewska A, Nightingale MS, Moss J (1992). Molecular characterization of NAD:arginine ADP-ribosyltransferase from rabbit skeletal muscle. Proc Natl Acad Sci USA.

[B12] Okazaki IJ, Zolkiewska A, Nightingale MS, Moss J (1994). Immunological and structural conservation of mammalian skeletal muscle glycosylphosphatidylinositol-linked ADP-ribosyltransferases. Biochemistry.

[B13] Okazaki IJ, Kim HJ, McElvaney NG, Lesma E, Moss J (1996). Molecular characterization of a glycosylphosphatidylinositol-linked ADP-ribosyltransferase from lymphocytes. Blood.

[B14] Glowacki G, Braren R, Cetkovic-Cvrlje M, Leiter EH, Haag F, Koch-Nolte F (2001). Structure, chromosomal localization, and expression of the gene for mouse ecto-mono(ADP-ribosyl)transferase ART5. Gene.

[B15] Zolkiewska A, Moss J (1993). Integrin alpha 7 as substrate for a glycosylphosphatidylinositol-anchored ADP-ribosyltransferase on the surface of skeletal muscle cells. J Biol Chem.

[B16] Zolkiewska A, Moss J (1995). Processing of ADP-ribosylated integrin alpha 7 in skeletal muscle myotubes. J Biol Chem.

[B17] Burkin DJ, Kaufman SJ (1999). The alpha7beta1 integrin in muscle development and disease. Cell Tissue Res.

[B18] Mark H von der, Williams I, Wendler O, Sorokin L, Mark K von der, Pöschl E (2002). Alternative splice variants of alpha 7 beta 1 integrin selectively recognize different laminin isoforms. J Biol Chem.

[B19] Zhao Z, Gruszczynska-Biegala J, Zolkiewska A (2005). ADP-ribosylation of integrin alpha7 modulates the binding of integrin alpha7beta1 to laminin. Biochem J.

[B20] Zolkiewska A (2005). Ecto-ADP-ribose transferases: cell-surface response to local tissue injury. Physiology (Bethesda).

[B21] Olson EN (1990). MyoD family: a paradigm for development?. Genes Dev.

[B22] Weintraub H, Dwarki VJ, Verma I, Davis R, Hollenberg S, Snider L, Lassar A, Tapscott SJ (1991). Muscle-specific transcriptional activation by MyoD. Genes Dev.

[B23] Wright WE, Sassoon DA, Lin VK (1989). Myogenin, a factor regulating myogenesis, has a domain homologous to MyoD. Cell.

[B24] Braun T, Bober E, Buschhausen-Denker G, Kohtz S, Grzeschik KH, Arnold HH (1989). Differential expression of myogenic determination genes in muscle cells: possible autoactivation by the Myf gene products. EMBO J.

[B25] Pollock R, Treisman R (1991). Human SRF-related proteins: DNA-binding properties and potential regulatory targets. Genes Dev.

[B26] Gossett LA, Kelvin DJ, Sternberg EA, Olson EN (1989). A new myocyte-specific enhancer-binding factor that recognizes a conserved element associated with multiple muscle-specific genes. Mol Cell Biol.

[B27] Wright WE, Binder M, Funk W (1991). Cyclic amplification and selection of targets (CASTing) for the myogenin consensus binding site. Mol Cell Biol.

[B28] Buskin JN, Hauschka SD (1989). Identification of a myocyte nuclear factor that binds to the muscle-specific enhancer of the mouse muscle creatine kinase gene. Mol Cell Biol.

[B29] Brennan TJ, Olson EN (1990). Myogenin resides in the nucleus and acquires high affinity for a conserved enhancer element on heterodimerization. Genes Dev.

[B30] Black BL, Olson EN (1998). Transcriptional control of muscle development by myocyte enhancer factor-2 (MEF2) proteins. Annu Rev Cell Dev Biol.

[B31] Grahnert A, Friedrich M, Pfister M, Haag F, Koch-Nolte F, Hauschildt S (2002). Mono-ADP-ribosyltransferases in human monocytes: regulation by lipopolysaccharide. Biochem J.

[B32] Friedrich M, Grahnert A, Paasch U, Tannapfel A, Koch-Nolte F, Hauschildt S (2006). Expression of toxin-related human mono-ADP-ribosyltransferase 3 in human testes. Asian J Androl.

[B33] Krause K, Wasner M, Reinhard W, Haugwitz U, Dohna CL, Mossner J, Engeland K (2000). The tumour suppressor protein p53 can repress transcription of cyclin B. Nucleic Acids Res.

[B34] Young TL, Santella RM (1988). Development of techniques to monitor for exposure to vinyl chloride: monoclonal antibodies to ethenoadenosine and ethenocytidine. Carcinogenesis.

[B35] Boyd KE, Wells J, Gutman J, Bartley SM, Farnham PJ (1998). c-Myc target gene specificity is determined by a post-DNAbinding mechanism. Proc Natl Acad Sci USA.

[B36] Altschul SF, Gish W, Miller W, Myers EW, Lipman DJ (1990). Basic local alignment search tool. J Mol Biol.

[B37] Thompson JD, Higgins DG, Gibson TJ (1994). CLUSTAL W: improving the sensitivity of progressive multiple sequence alignment through sequence weighting, position-specific gap penalties and weight matrix choice. Nucleic Acids Res.

[B38] Baxevanis AD (2002). Modeling Structure from Sequence. Current Protocols in Bioinformatics.

[B39] Lassar AB, Buskin JN, Lockshon D, Davis RL, Apone S, Hauschka SD, Weintraub H (1989). MyoD is a sequence-specific DNA binding protein requiring a region of myc homology to bind to the muscle creatine kinase enhancer. Cell.

[B40] Rudnicki MA, Schnegelsberg PN, Stead RH, Braun T, Arnold HH, Jaenisch R (1993). MyoD or Myf-5 is required for the formation of skeletal muscle. Cell.

[B41] Hasty P, Bradley A, Morris JH, Edmondson DG, Venuti JM, Olson EN, Klein WH (1993). Muscle deficiency and neonatal death in mice with a targeted mutation in the myogenin gene. Nature.

[B42] Nabeshima Y, Hanaoka K, Hayasaka M, Esumi E, Li S, Nonaka I, Nabeshima Y (1993). Myogenin gene disruption results in perinatal lethality because of severe muscle defect. Nature.

[B43] Li H, Capetanaki Y (1993). Regulation of the mouse desmin gene: transactivated by MyoD, myogenin, MRF4 and Myf5. Nucleic Acids Res.

[B44] Fickett JW (1996). Quantitative discrimination of MEF2 sites. Mol Cell Biol.

[B45] Ryseck RP, Macdonald-Bravo H, Mattei MG, Ruppert S, Bravo R (1989). Structure, mapping and expression of a growth factor inducible gene encoding a putative nuclear hormonal binding receptor. EMBO J.

[B46] Corda D, Di Girolamo M (2003). Functional aspects of protein mono-ADP-ribosylation. EMBO J.

[B47] Di Girolamo M, Dani N, Stilla A, Corda D (2005). Physiological relevance of the endogenous mono(ADP-ribosyl)ation of cellular proteins. FEBS J.

[B48] Tapscott SJ, Davis RL, Thayer MJ, Cheng PF, Weintraub H, Lassar AB (1988). MyoD1: a nuclear phosphoprotein requiring a Myc homology region to convert fibroblasts to myoblasts. Science.

[B49] Yaffe D, Saxel O (1977). Serial passaging and differentiation of myogenic cells isolated from dystrophic mouse muscle. Nature.

[B50] Li Z, Paulin D (1993). Different factors interact with myoblast-specific and myotube-specific enhancer regions of the human desmin gene. J Biol Chem.

[B51] Li Z, Paulin D (1991). High level desmin expression depends on a muscle-specific enhancer. J Biol Chem.

[B52] Horlick RA, Benfield PA (1989). The upstream muscle-specific enhancer of the rat muscle creatine kinase gene is composed of multiple elements. Mol Cell Biol.

[B53] Naidu PS, Ludolph DC, To RQ, Hinterberger TJ, Konieczny SF (1995). Myogenin and MEF2 function synergistically to activate the MRF4 promoter during myogenesis. Mol Cell Biol.

[B54] Molkentin JD, Black BL, Martin JF, Olson EN (1995). Cooperative activation of muscle gene expression by MEF2 and myogenic bHLH proteins. Cell.

[B55] Black BL, Molkentin JD, Olson EN (1998). Multiple roles for the MyoD basic region in transmission of transcriptional activation signals and interaction with MEF2. Mol Cell Biol.

[B56] Fickett JW (1996). Coordinate positioning of MEF2 and myogenin binding sites. Gene.

[B57] Kim UH, Kim MK, Kim JS, Han MK, Park BH, Kim HR (1993). Purification and characterization of NAD glycohydrolase from rabbit erythrocytes. Arch Biochem Biophys.

[B58] Braren R, Glowacki G, Nissen M, Haag F, Koch-Nolte F (1998). Molecular characterization and expression of the gene for mouse NAD+:arginine ecto-mono(ADP-ribosyl)transferase, Art1. Biochem J.

