# Supplementary material for: Identification of two regulatory binding sites which confer myotube specific expression of the mono-ADP-ribosyltransferase ART1 gene
Source: BMC Mol Biol. 2008 Oct 21;9:91. doi: 10.1186/1471-2199-9-91 (PMC2575215; doi:10.1186/1471-2199-9-91)
Supplement: Additional file 1 — The long fragment of the mouse ART1 proximal promoter contains four additional cis-regulatory consensus sites, putative candidates for enhancing muscle specific gene expression. Genomic sequences of the ART1 proximal promoter from mouse (NT_009237.17) were analysed using the Transcription Element Search System (TESS) program [39]. The numbering of the nucleotides refers to the nucleotide position -323 to -1236 relative to the putative transcription start site (numbered with +1). Putative cis-elements are indicated by black boxes with sequences in white. [file 1471-2199-9-91-S1.pdf]

```
-1236                                                     CTTTCTCTCGCACC
                    A/T-rich
-1222  TCCCTTGTGCTTGACTATAAATAACCTAGTTCTGAGTTCAGATGCCCAGTTGGGTGTAAT

-1162  GGGAGAAGGACCCAGCTTGCGGGAAGGTGAAGGAGGGACGTTCTTAACACCAAGGGTTAG
                E box
-1102  AGATGCCTCCTGTTGGGGCCACCATTAGCATAGGATAAGGCCTCTCCAGTCCAGCTCATG

-1042  AATTTACCCATCACCACCCTTTACCACCTCATATTGTCAGCCAAATAAAGTTTCCTTCAC

 -982  CCAAAAGCCTCTTTTCTCCTCCAATCCTTCCTGAAGGCTAAAGAGAAAAACAAACTATGT

 -922  CCAGCAGTGATCCCTTCCTTCCCTCCTGACCCCATCCTCCCTTTCTGGGAGGAATTCAGG
                             E box
 -862  TACTTGCAGTTTCTGGGACAGCAGATGCTGTGTGGGGGCAGCCTATGGCTTGGGGATTTT
                                                      E box
 -802  CTTATATCCTAGCACCCGGAACTCCAAGAAGTGGAGTCAAATCTGGTCACGTGTGGGTCA

 -742  GGGATCCTGAGAGGGGCAGCATCCTCTCAGGTCACACGGTGAGTCAGAGATCCAGCAGGA

 -682  AGCTAGGTTCTTTAGGGCAGAACAAAGCTTGCCCTAAACAACATGCATTGTAAACTTTAT

 -622  ATGCCCCAGTACAGGGGAACTTCGGGGCCAAGAAGTGGGAGTGGGTGGGTAGGGGAGCAG

 -562  GGTGGTGGTGGGATATAGGGGGCTTTCGGGATAGCATTTGAAATGTAAATGAAGAAAATA

 -502  CCTAATAAAAATTTGAAAAAAAAAAAAAAAAAAAAAAAAAAAGAACATGCATTGTGGTGG

 -442  TACATGCCTATAACCCTAGCAATCGGCAGGGAAGCTGGGGCAACAGGATTGCTTCAAGAT

 -382  CAAGGTCAGCCTGGACTTTGTGAAAGAAAGTATTAAGACAACAACAACAATTAACCACCA
```
